# The Rapeseed Oil Based Organofunctional Silane for Stainless Steel Protective Coatings

**DOI:** 10.3390/ma13102212

**Published:** 2020-05-12

**Authors:** Karol Szubert, Jarosław Wojciechowski, Łukasz Majchrzycki, Wojciech Jurczak, Grzegorz Lota, Hieronim Maciejewski

**Affiliations:** 1 Faculty of Chemistry, Adam Mickiewicz University in Poznan, Uniwersytetu Poznańskiego 8, 61-614 Poznan, Poland; maciejm@amu.edu.pl; 2 Institute of Chemistry and Technical Electrochemistry, Poznan University of Technology, Berdychowo 4, 60-965 Poznan, Poland; jaroslaw.g.wojciechowski@put.poznan.pl (J.W.); grzegorz.lota@put.poznan.pl (G.L.); 3 Centre for Advanced Technologies, Adam Mickiewicz University in Poznan, Uniwersytetu Poznańskiego 10, 61-614 Poznan, Poland; lukmaj@amu.edu.pl; 4 Faculty of Mechanical and Electrical Engineering, Polish Naval Academy, Śmidowicza 69, 81-127 Gdynia, Poland; w.jurczak@amw.gdynia.pl

**Keywords:** renewable resources, rapeseed oil, sol-gel processes, corrosion, stainless steel

## Abstract

The earlier obtained organosilicon derivatives of rapeseed oil were used for the production of coatings protecting steel surface against corrosion. Vegetable oils have been hitherto used for temporary protection of metals against corrosion, while thanks to the synthesis of appropriate organosilicon derivatives, it is now possible to create durable protective coatings. Due to the presence of alkoxysilyl groups and the use of the sol-gel process, the coatings obtained were bonded to the steel surface. The effectiveness of the coatings was checked by electrochemical methods and steel surface analysis.

## 1. Introduction

Stainless steel is widely used in many areas of industry, mainly because of its mechanical properties and resistance to corrosion. However, in certain conditions, e.g., in the presence of halogen ions, corrosion may appear. Taking into account the increasingly restrictive regulations of the environment protection, the use of popular corrosion inhibitors based on phosphates, chromates and other heavy metals has been much restricted. In response, new alternative anticorrosion agents have been proposed. For instance, the high effectiveness of coatings based on organosilicon compounds has been evidenced [1,2,3,4,5,6,7,8,9,10]. The use of silanes for metal surface treatment has been also found to improve the adhesiveness of paints [11,12].

The use of sol-gel processes for the protection of metal surfaces has been presented in the literature quite extensively, for example, see References [1,2,7,13,14,15,16]. Publications describe the anticorrosive properties of coatings based on compounds, including tetraethoxysilane, octyltriethoxysilane, (3-mercaptopropyl)trimethoxysilane1,2-bis(trimethoxysilyl)ethane, (3-aminopropyl) triethoxysilane, triethoxysilane and others [1,2,3,4,5,6,7,8,9,10,11,12,13,14,15,16,17], have appeared over the past several decades. Aqueous and alcoholic organosilicon compound solutions were used. Generally, the siloxane coatings are a physical barrier to aggressive electrolyte solutions in protecting metal surfaces. The highly cross-linked interfacial layer developed in the sol-gel system retards the transport of corrosion factors and products. In addition, the siloxane layer can also effectively blocks cathodic sites on the metal surface, due to the formation of metal-O-Si covalent bonds at the interface [2,18]. 

Hydrolysis and condensation reactions take place in the sol-gel solutions [1,2,19,20,21,22]. In the presence of water silanol groups are formed via hydrolysis reaction. The following condensation reactions between the formed silanol groups (Si-OH) and alkoxy groups (Si-O-R) lead to crosslinked siloxane (Si-O-Si). The silanol groups (SiOH) can also react with the metal hydroxyl groups (metal-OH) present on the metal surface via the formation of covalent metal-O-Si bonds [1,2]. Therefore, a properly prepared surface of the base material should contain a large number of reactive hydroxyl groups.

Increasingly stringent regulations following from the natural environment concerns and the search for ‘green’ corrosion inhibitors have stimulated the interest in natural products that could be used as anti-corrosive inhibitors. In recent investigations attempts have been made to replace crude oil products with the compounds from renewable sources [23,24,25,26]. The use of materials based on vegetable oils is particularly beneficial because of their low cost, high availability and low ecotoxicity. 

So far, vegetable oils have been used for temporary anticorrosive protection of metals. On the metal surface, they form a thick, relatively soft and impermanent coating providing a barrier effect. They are cheap and easy to use, but require careful purification of the metal surface prior to application and can be ineffective, especially when used for a long time [27].

In this work, we would like to present the anti-corrosive properties of rapeseed oil-based organofunctional silane coatings deposited on the surface of 304 stainless steel. From the chemical point of view, the long aliphatic chains present in vegetable oils can be applied for the synthesis of new silanes and polysiloxanes with hydrophobic properties that are attractive materials for producing coatings protecting against the adverse effects of water and moisture [28]. Due to the presence of alkoxysilyl groups and the use of sol-gel process, the coatings obtained were bonded to the steel surface. The effectiveness of the coatings was checked by electrochemical methods and steel surface analysis.

## 2. Materials and Methods 

The chemicals were obtained from Sigma-Aldrich and used without any additional preparatory steps. The 304 stainless steel discs (2.79 cm in diameter) with the following nominal composition: max 0.015 wt% S, max 0.045 wt% P, max 0.07 wt% C, max 0.11 wt% N, max 1.00 wt% Si, max 2.00 wt% Mn, 8.00 wt%–10.50 wt% Ni and 17.50 wt%–19.50 wt% Cr; were purchased from Rowitex Ltd. Company. The rapeseed oil-based silane (RPTMS) was synthesized according to the procedure described earlier [28].

### 2.1. Treatment of 304 Stainless Steel Surface

The surfaces of 304 stainless steel discs were subjected to a two-stage process of chemical purification. At first, the discs were degreased by acetone in an ultrasonic bath (10 min) and then they were immersed in a hot 10% solution of KOH (85 °C, 15 min) to unblock hydroxyl groups on the stainless steel surface. Finally, the discs were washed with distilled water and dried at 80 °C (30 min). Then a series of three solutions of silane RPTMS in ethanol was prepared as specified in Table 1.

Acetic acid was added in the amount indispensable for adjusting the pH of the solutions to the value of about 4. The measured pH values were about 3.8–3.9. The solutions were stirred for 3 h, except for the solutions R3 and R5 that were stirred for 72 h. After the stirring was completed, the samples of stainless steel discs were fully submerged in the solutions for 5 min. In the end, all the samples were cured at 80 °C for 1 h. 

### 2.2. Physicochemical and Surface Morphology Analysis

The pH values of the solutions R1–5 were measured using a pH meter FiveEasy F20 manufactured by Mettler-Toledo GmbH equipped with an electrode LE438. Before the measurements, the pH meter was calibrated.

Scanning electron microscopy (SEM) images were taken on an FEI Quanta 250 FEG microscope equipped with an EDAX Energy Dispersive Spectroscopy detector (EDS). The images were taken in high vacuum mode with 10 kV accelerating voltage. EDS mapping was realized with an electron beam energy of 20 keV using an EDS Octane SDD detector (EDAX). The steel samples were prepared for SEM imaging by gluing onto the standard SEM carbon adhesive tape. 

In order to estimate the thickness of the RPTMS coating, the steel covered with it was scratched with a sharp edge of an aluminum sheet. As the aluminum hardness is lower than that of steel, it is reasonable to assume that only the RPTMS coating was destroyed by a scratch. Afterward, the scratched region was examined using an atomic force microscope (Agilent 5500). Images from a scan area of 50 µm x 50 µm were flattened using a 2nd order polynomial to minimize the sample slope and bowl from the scanner. From each AFM image, 10 profiles across the scratch were extracted. All profiles were fitted by step curves, which allows the proper determination of scratch depth, along with the sample roughness. Finally, the mean value and standard deviation of so-obtained step height values were calculated.

Fourier transform infrared (FT-IR) spectra were recorded on a Bruker Tensor 27 Fourier transform spectrometer equipped with a SPECAC Golden Gate diamond ATR (attenuated total reflection) unit. In each case, 16 scans were collected for a spectrum at the resolution of 2 cm^−1^. Measurements were carried out at three sites for each sample and the spectra were averaged using the OPUS Data Collection Program. Static water contact angle (WCA) measurements on all samples were made using a Krüss GmbH DSA 100 Expert Drop Shape Analyzer equipped with a software-controlled (DAS4 2.0): x, y, z-axis table, quadruple dosing unit with zoom and focus adjustment, illumination, and a camera with 780 x 580 px resolution. All presented data are arithmetic means from measurements made for 5 drops per sample. The measurements of contact angles were performed immediately after the deposition of a drop on a studied surface and the measurement time was about 1 s.

### 2.3. Electrochemical Measurements

Electrochemical measurements were performed in a three-electrode Plexiglas^®^ cell system. Saturated calomel electrode (SCE) and a platinum disc served as reference and counter electrodes, respectively. The 304 stainless steel samples with and without the deposited RPTMS layers were used as working electrodes.

An electrochemical impedance spectroscopy (EIS) study was carried out at a frequency range from 100 kHz to 10 mHz. The amplitude of the applied signal was ±10 mV versus open circuit potential (OCP). Additionally, potentiodynamic polarization (PP) tests were performed. The working electrode was polarized cathodically (-250 mV vs. OCP) and then anodically until current values of 100 µA were reached. The scan rate was equal to 0.2 mV s^−1^. All the electrochemical tests were performed in 3.5% NaCl (Sigma Aldrich) solution at ambient conditions using an electrochemical workstation potentiostat/galvanostat VMP3 (Biologic, France) with an impedance module. The analysis of the electrochemical test results was carried out using the EC-Lab^®^ software.

## 3. Results

### 3.1. Physicochemical and Surface Morphology Analysis

**Figure 1:**
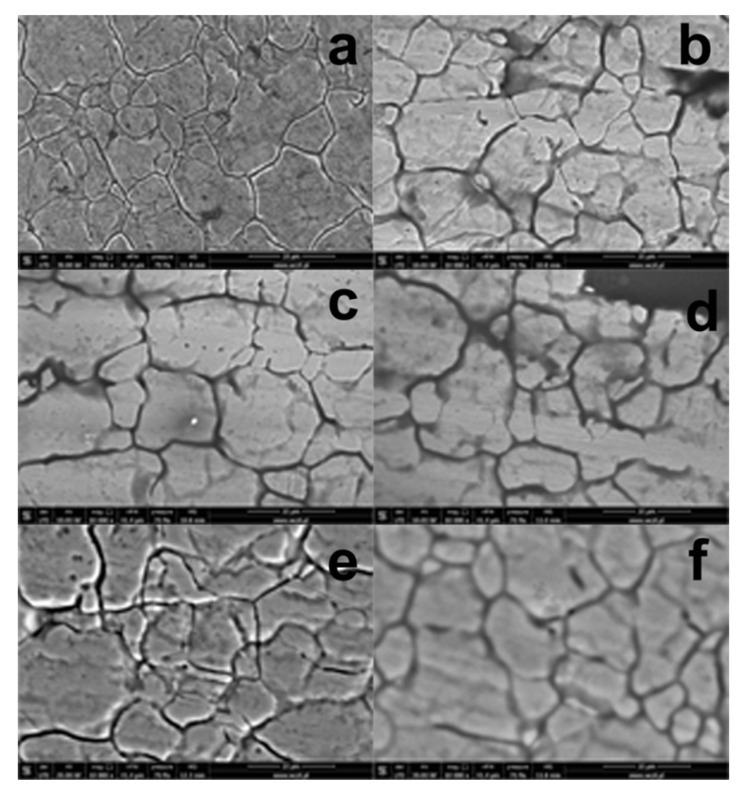
Scanning electron microscope (SEM Hitachi, SU3500) image of (**a**) 304 stainless steel after acetone and hot 10% KOH solution treatment; (**b**) sample R1; (**c**) sample R2; (**d**) sample R3; (**e**) sample R4; (**f**) sample R5 at magnification 10k x.

**Figure 2:**
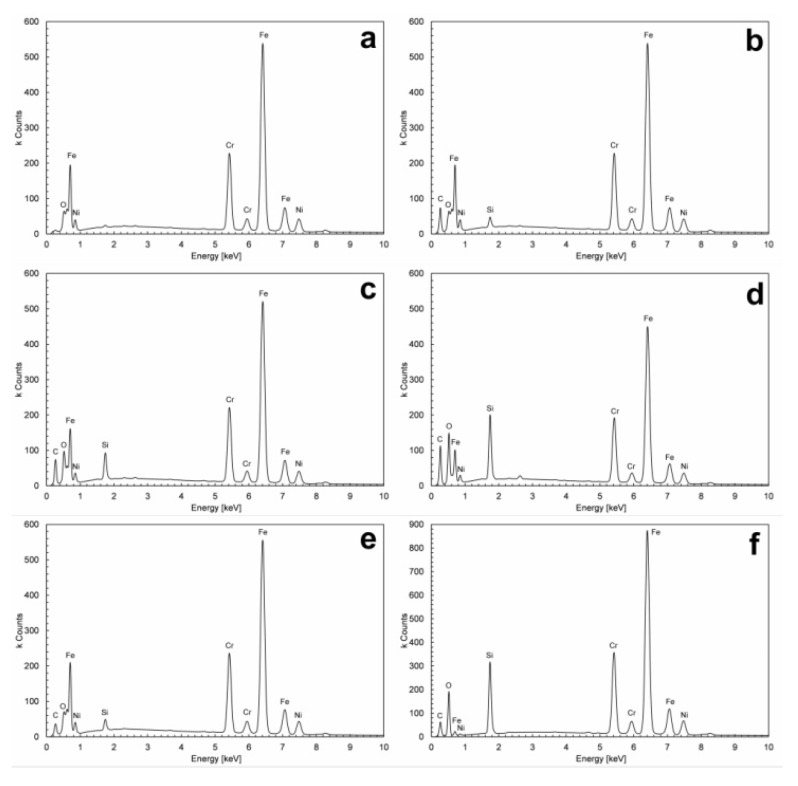
Energy-dispersive X-ray spectroscopy (EDS) results of (**a**) 304 stainless steel after acetone and hot 10% KOH solution treatment; (**b**) sample R1; (**c**) sample R2; (**d**) sample R3; (**e**) sample R4; (**f**) sample R5.

Figure 1a presents an SEM image of 304 stainless steel, degreased with acetone and chemically activated in a KOH solution. The image shows grains and crackings on the steel surface, typical of austenitic steel [29], which confirms partial etching of the steel surface taking place upon such a chemical activation. The EDS spectrum of the purified steel (Figure 2a) shows signals assigned to iron, chromium and nickel, while the signal attributed to carbon atoms is very weak, confirming total removal of organic impurities from the steel surface. 

The steel plates prepared in this way were used for testing the anticorrosive coatings. The coatings were obtained in the sol-gel process from alcohol solutions of RPTMS silane. As a result of hydrolysis and condensation processes, taking place in the prepared sol-gel solutions, a well-developed linked network of Si-O-Si covalent bonds was obtained. The process of condensation between the -OH groups occurring on the steel surface and the Si-OH and Si-O-CH_3_ groups present in the solution led to the formation of hydrolytically stable covalent metal-O-Si bonds, through which the coating was firmly attached to the surface. Moreover, the long aliphatic chain (coming from the oil) bonded to the silicon atom endowed the steel surface with hydrophobic character. 

Figure 1b–f presents SEM images of the steel surface coated with RPTMS-based material. These images are identical to those from the SEM images of bare steel (Figure 1a). The generation of the protective coatings on the steel surface did not produce significant changes in the steel plates surface; all samples studied were characterized by cracked surface layers. Only for a few coated steel plates (see Figure 1d,f) the metal surface image was blurred, which was most probably due to partial dispersion of electrons by the siloxane layer formed on the metal surface. The generation of an additional thin organometallic coating on the steel surface was confirmed by the EDS spectra (Figure 2b–f) showing additional signals coming from carbon and silicon atoms. The highest intensities of the signals assigned to silicon were recorded for the coatings deposited from the solutions containing TEOS and stirred for 72 h (R3 and R5). Extension of the stirring time of the solutions had a beneficial effect on the hydrolysis and condensation of TEOS, while the coatings obtained in such conditions were richer in silicon. Unfortunately, the extended time of stirring led also to a partial condensation of RPTMS, (the polysiloxane was responsible for the solution turbidity and after cessation of stirring the system became stratified), which limited its contribution in coating formation on the steel surface.

EDS mapping was performed and analyzed in order to check the uniformity of carbon and silicon distribution in the coatings made (results are shown in the Supplementary Materials). According to the EDS mapping, in the coatings R1–R4, the elements C and Si were uniformly distributed over the metal surface (no dark areas or spots in the images that would indicate the fragments of steel not covered with the coating). Only in the holes in the steel surface the accumulation of C and Si atoms was observed (brighter areas in the images). Single dark spots were observed in the images of EDS mapping of Si for coating R5, marking the areas without these atoms, which indicates inhomogeneity of the coating.

**Figure 3:**
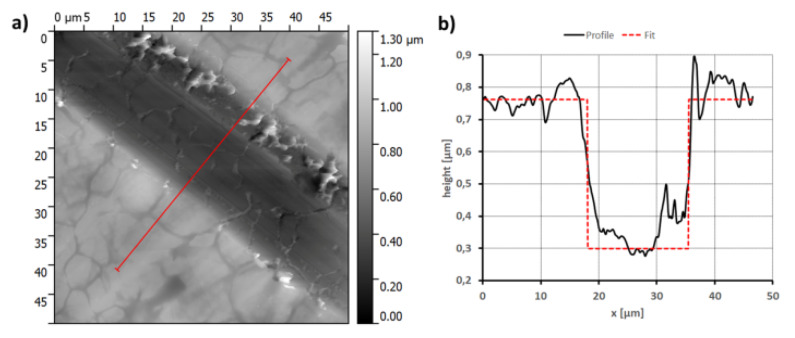
(**a**) Typical AFM image of the scratched layer, (**b**) extracted profile with fitted step curve.

As has been mentioned, in order to determine the thickness of the deposited RPTMS coatings, the steel substrates were scratched by a sharp edge of an aluminum sheet. The depth of the scratches was measured by an atomic force microscope (AFM). A typical result with the extracted profile is shown in Figure 3. Finally, the mean value and standard deviation of such obtained step height values were calculated. The results of the thickness of such analyzed layers are presented in Table 2. 

The thickest coating was R2 obtained from the solution containing 20%wt of RPTMS and TEOS subjected to stirring for 3 h. The extension of the time of the solution stirring (coating R3) had an adverse effect on the thickness of the coating. Our previous studies [28] have shown that after 3 h, the highest concentration of RPTMS hydrolysis products was obtained, after 72 h of stirring, polysiloxanes dominate in the solution. In addition, it should be mentioned that in the case of the R2 coating, a higher RPTMS concentration was used, which may also affect the thickness of the obtained coating.

**Figure 4:**
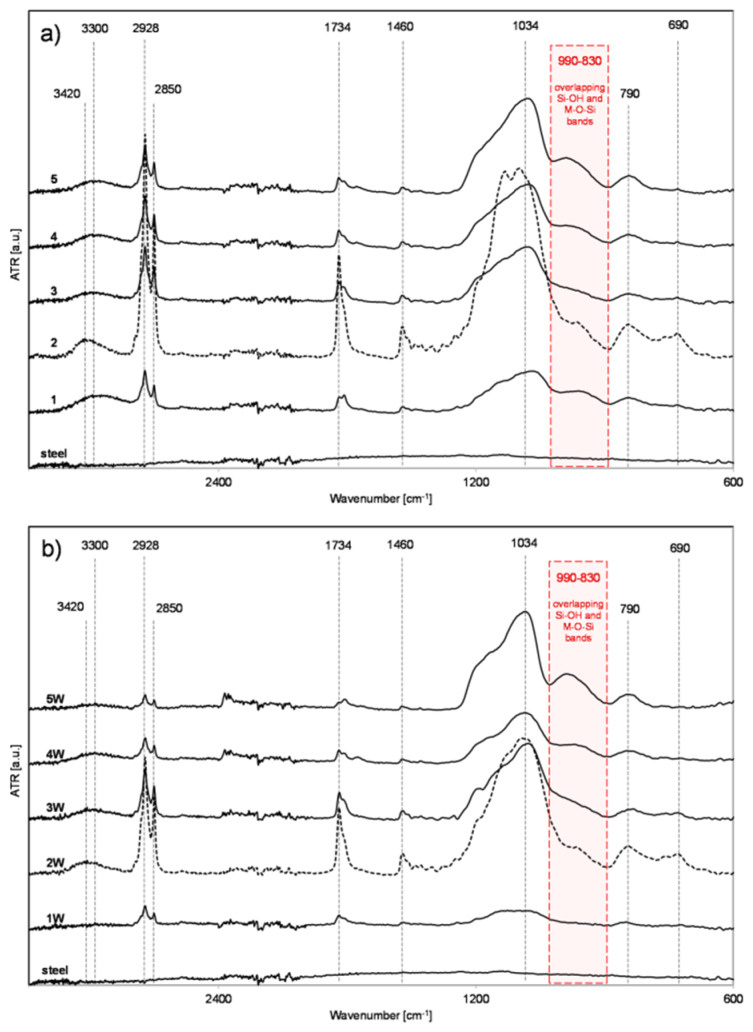
(**a**) FT-IR spectra of coated steel samples and (**b**) corresponding samples after conditioning in water.

Freshly coated steel samples, as well as the corresponding samples conditioned for 24 h in distilled water, were subjected to the FT-IR analysis to evaluate the influence of the silane solutions composition and the time of condensation process applied prior to their deposition on the steel surface, on the quality of coatings formed and their durability. The durability evaluation of the coatings formed was based on the comparison of intensities of the characteristic bands observed in the FT-IR spectra of measured samples before and after their conditioning in water. The characteristic bands observed in the spectra of all tested samples, presented in Figure 4 confirm the presence of expected functional groups on their surface. Broad bands with maxima at 3300 or 3420 and probably these in the range of 980–830 cm^−1^ observed in the spectra should be attributed to the presence of silanol (Si-OH) groups in the structure of formed coatings. The bands at 2928, 2850 and 1460 cm^−1^ result from the stretching asymmetric, symmetric and scissoring vibrations, respectively, of C-H bonds present in methylene (-CH_2_-) moieties of long alkyl chains. The presence of carbonyl groups is confirmed by the presence of a band at 1734 cm^−1^ characteristic of the stretching vibrations of the C=O bond. The broad band in the range of 1250–1000 cm^−1^ is a result of overlapping vibrations characteristic of Si-O-Si, Si-OCH_3_ and C-O bonds. The formation of Si-O-Si linkages of different geometry is also confirmed by the presence of bands with maxima at 790 and 690 cm^−1^.

In the case of the discussed systems, the presence of bands characteristic for M-O-Si bonds is also expected. Literature data indicate that the occurrence of this type of band can be expected in the range of 1000-900 cm-1 [30]. More precisely Seyedmonir et al. assigned the band at 925 cm^−1^ to the of Mo-O-Si moiety [31], Naviroj et al. attributed weak spectral bands near 963 and 950 cm^−1^ to Al-O-Si and Ti-O-Si linkages, respectively [32], Miller and Ishida attributed the antisymmetric stretching vibration near 965 cm^−1^ to the Pb-O-Si bonding in plumbosiloxane [33]. Bands occurring near 960 cm^−1^ have been also attributed to the Ni-O-Si and Ti-O-Si linkages by Saadi et al. and Sayari et al. respectively [34,35]. Zeitler and Brown in their much earlier research attributed absorption bands at 926 and 919 cm^−1^ to the Ti-O-Si bonds in tetrakistriphenylsiloxy- and tetrakistrimethylsiloxytitanium derivatives respectively [36].

In light of the cited data, the broad band observed in the spectra of the measured sample in the range of 980 to 830 cm^−1^ could be attributed to the M-O-Si bonds vibrations instead of Si-OH ones as it was mentioned above. Nevertheless, considering the nature of measures samples, the method of their preparation and the specifics of the analytical technique applied (FT-IR in ATR mode) such an assignment would be an overinterpretation. Most likely the presence of this band is a result of the overlapping vibrations of Si-OH and M-O-Si bonds.

It can be clearly seen that the relative intensities of the above-mentioned bands differ significantly in each spectrum, which confirms that the composition of the silane solutions, as well as their hydrolysis and condensation period strongly, influence the quality of coatings formed. The highest intensities of characteristic bands were observed in the spectrum of sample R2 that also confirms that the layer formed on its surface is the thickest of all measured samples. It is related to the highest concentration of silanes in the solution used for its preparation. The intensity of the spectrum recorded for sample R3, prepared from a solution of silane of the same concentration (see Table 1) but subjected to the prolonged to 72 h hydrolytic polycondensation process is significantly lower. It can be explained by the formation of poorly soluble, high-molecular species during prolonged polycondensation process, resulting in solution inhomogeneity and subsequently lower sample surface loading.

A comparison of the intensities of the spectra recorded for fresh samples (Figure 4a) with those subjected to aging in water (Figure 4b) also suggest the highest quality of coating formed on the surface of sample R2. The intensity of conditioned sample R2W (after immersion in water) is lower than this recorded for sample R2 but still the highest of all soaked samples. However, it should be also noted that the intensity of the spectrum of sample R3W after its immersion in water is almost unchanged in comparison to that of sample R3 recorded before immersion in water. A significant decrease in the intensity of the bands observed in the spectra of samples R1W, R4W, and R5W as well as a change in the relative intensity ratio of 2928 and 2850 cm^−1^ bands to the 1250–1000 cm^−1^ one is a result of RPTMS leaching. This effect is the most pronounced for sample R1 and R1W coated with the use of the solution containing only RPTMS with no addition of TEOS. The results of FT-IR analysis suggest that the application of the silane solution of higher RPTMS concentration and containing TEOS permits the formation of more durable (hydrolytically stable) coatings, probably of better barrier properties. 

Table 3 presents the values of water contact angles (WCA) at the purified bare and modified 304 stainless steel surface. As follows from the measured WCA values, the modified stainless steel surface is hydrophilic, as its WCA is below 90°. The silane-based coatings generated on the 304 stainless steel surface endow this surface with hydrophobic character (R1, R2, R4). The surface hydrophobicity is attributed to the effect of long aliphatic chains from RPTMS silane. The steel surfaces covered with R3 and R5 coatings obtained from the solutions stirred for 72 h are hydrophilic as their WCA are lower than 90°. The pure TEOS-based materials (silica, coatings) are hydrophilic [37,38]. As mentioned earlier, the longer stirring of the solutions promotes the condensation reaction of RPTMS; the contribution of long aliphatic chains in the obtained coating is lower, which may affect lower WCA values.

### 3.2. Electrochemical Measurements

**Figure 5:**
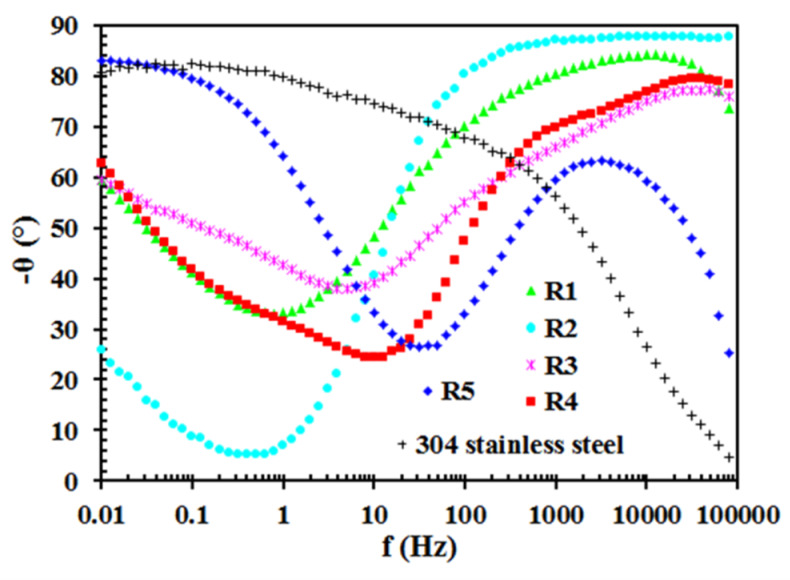
Bode plots (phase angle vs. frequency) of 304 stainless steel and samples R1–5.

**Figure 6:**
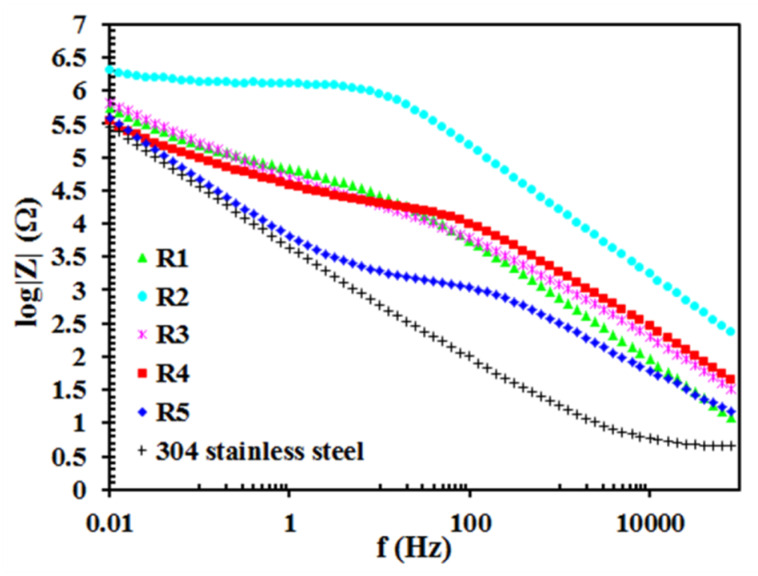
Bode plots (impedance modulus vs. frequency) of 304 stainless steel and samples R1–5.

Figure 5 and Figure 6 show the results of EIS measurements. These are Bode plots, i.e., the relationship of the phase angle and the impedance modulus from the signal frequency. The obtained results confirm partly the physicochemical and surface morphology analysis. In the range of high-frequency values, sample R2 shows the lowest phase angle (θ), which is equal to almost –90°. This indicates that the coating deposited on sample R2 is the thickest or/and is the most uniform, i.e., it shows the best dielectric properties [39,40,41,42,43,44]. Additionally, in the range of low-frequency values, the phase angle for this sample is the highest. Such a large difference in θ, depending on the frequency, indicates that the two time constants describing the siloxane coating (first time constant) and the electrochemical reactions at the passive oxide film/electrolyte interface (second time constant) are very well separated [39,40,41,43]. This is another evidence confirming the thesis about the higher thickness and/or uniformity of the coating deposited on sample R2. As a consequence, the impedance modulus values for this sample are the highest in the whole range of the tested frequencies (Figure 6). The presence of a straight line with a slope of (-1) (Figure 6) indicates the presence of a constant phase element parameter, while the plateau - reflects the presence of a resistive element in the tested system [39,40]. As already mentioned, in the case of coatings with dielectric properties, which are deposited on metal and steel surfaces, the phase angle and impedance modulus depend not only on the thickness but also on the uniformity of the coating. If the coating is extremely porous and able to absorb a large amount of electrolyte solution, then conductive pathways are formed inside the coating and the phase angle values increase (high-frequency values). However, in this case, some convergence between the results of EIS tests (Bode tests) and the results of coating thickness measurements (AFM) should be noted. This applies to samples R2, R3 and R5. The only exception is R4 sample, which is also characterized by low phase angle values in the high-frequency range and is actually one of the thinnest. This probably indicates that this coating is very uniform, which will also be explained later when describing the results of potentiodynamic polarization tests. In turn, sample R5, whose coating was deposited from the solution containing the lowest RPTMS addition and the longest stirring time used, shows the highest phase angle values, which is associated with both lower thickness and worse uniformity. In the case of 304 stainless steel, the phase angle as well as the impedance modulus decrease and increase, respectively, as the frequency value decreases. Therefore, in this case, one time constant that describes the presence of an electrochemical reaction is observed [44]. In addition, the lack of a siloxane coating means that a plateau appears in the high frequency range (Figure 6). This is the detection of an electrolyte resistance. In the Bode plots (−θ vs f) obtained for sample R4, the presence of three loops (peaks) is observed including two (hardly visible ones) in the range of high-frequency values (~100 kHz–100 Hz). It does not necessarily indicate the presence of three time constants. Most likely, the appearance of the two above-mentioned loops (high-frequency values) is related to the siloxane coating itself and their presence is the result of the structure of this coating. Moreover, sample R5 is characterized only by one loop (peak) in high frequency values range. Although, the values of θ are in this case the highest. As mentioned above, the layers on samples R4 and R5 are one of the thinnest. In addition, both the stirring time of the solution and the amount of RPTMS additive influence this phenomenon. In each of these samples, a minimum amount of RPTMS was used. It is worth mentioning that the coatings deposited from solutions in which the greater amount of RPTMS was used are characterized by the highest values of the impedance modulus (Figure 6) in the range of the lowest frequency values.

**Figure 7:**
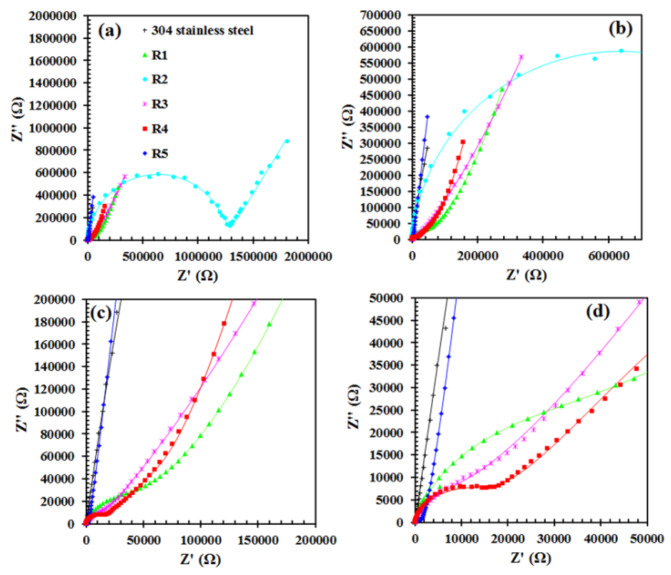
(**a**–**d**) Nyquist plots of 304 stainless steel and samples R1–5. Raw data (symbols) and fitted data (solid lines).

**Figure 8:**
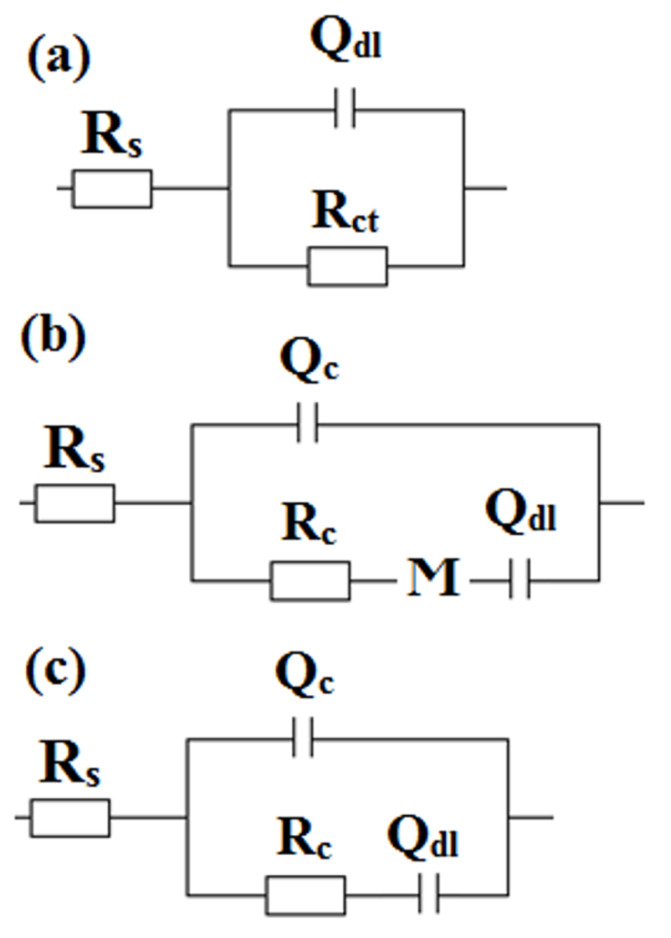
Electric equivalent circuits (EEC) fitted to EIS data of (**a**) bare 304 stainless steel, (**b**) samples R1, R3, R4, R5 and (**c**) R2.

Electrochemical impedance spectroscopy analysis allows matching electric equivalent circuits (EEC) to the tested electrochemical systems [39,40]. Figure 7 shows Nyquist plots. The symbols used to stand for the experimental data, while the lines stand for the data obtained as a result of EEC fitting. The degree of agreement between the lines and the experimental points informs about the correctness of the choice of EEC. Figure 8a–c shows the EEC that were fitted to the EIS test results of unmodified 304 stainless steel and samples R1–5. The elements constituting the presented circuits are resistors (R), constant phase element parameters (Q) and diffusion element (Ϻ): R_s_: uncompensated equivalent series resistance of the electrolyte solution, Q_dl_: constant phase element parameter describing electrical double layer, R_ct_: charge transfer resistance, Q_c_: constant phase element parameter describing siloxane coating, R_c_: siloxane coating resistance and Ϻ: an element that represents the restricted linear diffusion phenomena. It is worth mentioning that the constant phase element parameter (Q) replaces the capacitor, while the impedance of the constant phase element is given by [39,40,45,46]:

(1) $$Z\left(j\omega \right)=\frac{1}{Q{\left(j\omega \right)}^{\alpha }}$$

where j, ω and α are the imaginary numbers, angular frequency and factor reflecting capacitive dispersion, respectively. According to Equation (1), constant phase element expresses non-ideal capacitor behavior, i.e., it is a reflection of capacitive dispersion at the electrolyte/electrode interface and therefore parameter Q should no longer be identified as capacitance when α < 1. This is due to, among others, the chemical heterogeneity of the tested surface or even the adsorption of various types of ions. According to Yuan et al. [45] and Hirschorn et al. [46], there is an ability to asses effective capacitance of the coating as well as the electrical double layer on the basis of the constant phase element parameter and resistance values. However, in the further parts of the manuscript, the effective capacitance values are not compared, therefore they were not determined. In this situation, only the values of resistances and α for the particular constant phase element parameters are relevant for the description of the results of EIS tests. Thus, Q values are provided as F s^(α−1)^, which is consistent with Yuan et al. [45] and Hirschorn et al. [46]. 

On the surface of 304 stainless steel, which was subjected to electrochemical tests, there was a passive oxide film. According to Barsoukov and Macdonald [40], the selection of EEC for steels or metals containing passive oxide layers should take into account the constant phase element parameter and resistance of these layers, as well as the formation of an electrical double layer at the electrolyte/passive oxide film and passive oxide film/substrate element interfaces. Kocijan et al. [47] described the four most commonly used types of EEC taking into account two time constants and a single one that characterizes the presence of an oxide layer and electrical double layer at the passive oxide film/substrate element interface. Considering the work of Barsoukov and Macdonald [40], it was decided to use a circuit that contains elements describing the formation of an electric double layer at the electrolyte/passive oxide film interface. This is derived from the Nyquist plot that indicates the presence of one time constant. It should be the first possible time constant that is visible in the high-frequency range. In this case, only one time constant is visible over the entire frequency range. This is related to the methodology for preparing the steel surface. During the steel treatment, no mechanical cleaning was used. Additionally, etching in alkaline solutions allows for the removal of impurities, while preserving a passive oxide film on the steel surface. Therefore, already at the beginning of electrochemical tests, a passive oxide film was present on the surface of the steel sample. This is also confirmed by the results of EDS analysis (presence of oxygen). The use of two time constants usually concerns the situation when the oxide layer begins to form in the electrolyte solution in which the tests are carried out [47]. Then, in the Nyquist plots, in the range of high-frequency values, an outline of semi-circle appears, while on the Bode plots, more precisely in the graphs depicting dependence between phase angle and frequency, two loops appear, i.e., in the range of high- and low-frequency values.

The EEC for modified samples contains the constant phase element parameter (Q) and resistance (R) of siloxane coatings and electrochemical phenomena at the electrolyte/passive oxide film interface [44]. Siloxane coatings are porous, thus, they are able to absorb a certain amount of electrolyte. Therefore, this way of presenting the circuit is physically meaningful. The Ϻ element represents a particular diffusion phenomena i.e., a restricted linear diffusion with reflective boundary conditions [39,40,48,49,50,51]:

(2) $$Z\left(j\omega \right)=R_{d}\frac{\coth\left(\sqrt{\tau_{d}j\omega }\right)}{\sqrt{\tau_{d}j\omega }}$$

where R_d_ and τ_d_ are the diffusion resistance and diffusion time constant, respectively. τ_d_ is equal to L^2^/D, where L and D represent the sequence diffusion layer thickness and diffusion coefficient. Typically the Warburg impedance element, which specifies semi-infinite linear diffusion, is used to describe the diffusion phenomena occurring in electrochemical systems. The use of this element is justified if, in a certain frequency range, points on the Nyquist plot form a line whose slope is equal to (–1). If there are faradaic reactions in the tested electrochemical system, such a line usually appears in the medium and low-frequency range. However, in addition to the Warburg impedance element, there are other elements, which could be used to describe diffusion, including elements describing restricted linear diffusion (finite-length diffusion). In the case of EIS test results, the Ϻ element was used to describe the nature of individual samples with applied coatings. It is worth noting that all samples on which the coatings have been applied have a better or worse developed semi-circle in the high-frequency range. Then, in the range of medium-frequency values, the line adopts a diffusive character, while in the range of low-frequency values, it describes the presence of a constant phase element parameter. This situation, apart from the first time constant (high-frequency range), can be described by the presence of a diffusion element and a constant phase elements parameter. Therefore, in this situation, the most suitable choice is one that describes restricted linear diffusion. The nature of the Nyquist plots and the selection of Ϻ element is supported by, among others, Lasia [39] and Beguin and Frąckowiak [48]. Table 4 shows the values of all components of a chosen EEC. Sample R2, containing the thickest (AFM) siloxane coating is characterized by the highest value of the coating resistance (R_c_), which is tantamount to the best dielectric properties. The semi-circle in the range of high-frequency values shows the largest diameter (Figure 7a,b). Additionally, parameter Q is the lowest, while α is characterized by the highest value. In the medium- and low-frequency range, there is only a straight line whose slope corresponds to the presence of a constant phase element parameter. Therefore, the element describing the diffusion of mass is unnecessary. In other cases, i.e., when applying longer stirring times of the solution (72 h) and minimal addition of RPTMS, the coatings are thinner and less uniform, therefore the diffusion phenomena occur, which is associated with the presence of large, poorly soluble, high-molecular colloidal particles. However restricted linear diffusion with reflective boundary conditions model is mainly applied to the conductive polymers, thin passive oxide films, systems based on intercalation and insertion phenomena, thin solution films between the electrochemically active electrode and inactive surfaces [39,40,48,49,50,51]. Summarizing, restricted linear diffusion with reflective boundary conditions is based on the assumption that there is no concentration gradient, i.e., at x=L concentration gradient is equal to zero. Samples R1, 3, 4 and 5 indicate a typically diffusive character at intermediate frequencies (Figure 7). The shape of the Nyquist curves clearly indicates the model of restricted linear diffusion with reflective boundary conditions. This is certainly due to stainless steel and a passive oxide film presence. As already mentioned, the deposited coatings are porous, therefore the electrochemical corrosion process proceeds under the coating. A layer of corrosion products is formed on the surface of the passive oxide film. Over time, the coating delamination is in progress [18]. It can be stated that the oxide film builds up. The product of these processes is a layer that consists of metal oxides/hydroxides, ions and an electrolyte solution. It is a somewhat porous layer, filled with an electrolyte solution. First of all, it is a layer that conducts electric charge. Of course, the restricted linear diffusion model (reflective boundaries) is not an ideal model for this type of electrochemical system, however, at this point in time and at this stage of degradation of the surface of stainless steel, covered with this type of coating, this model can be used successfully and it is the best possible choice. As the electrolyte solution reacts, the impedance characteristics will change. The degradation of the coating will continue. Many more corrosion products will form under the coating. The coating will be filled with more electrolyte solution and more corrosion products in the form of ions and metal oxides/hydroxides. The impedance characteristic in the range of intermediate-frequency values will indicate the presence of diffusion phenomena, which then can be described by means of the Warburg impedance element or impedance element for restricted linear diffusion with transmissive boundary conditions [39,40,48]. Based on the results in Table 4, it can be concluded that the layers of corrosion products (under the coating) are relatively thin. This is evidenced by the low value of the diffusion time constant τ_d_ compared to the diffusion resistance R_d_. It is worth noting that the highest R_d_ value is indicated by sample R4, i.e., a sample that probably has an extremely uniform coating. The uniformity of the coating (R4) and its thickness (R2) affect the quantity and quality of corrosion products formed under the coating. The resulting layer of products is tighter. In addition, fewer ions and metal oxides/hydroxides appear in the layer as well as in its vicinity. Due to this, the diffusion process is characterized by high resistance (R4) or it can simply not be taken into account (R2). Considering the phenomenon of diffusion at the coating/passive oxide film interface, the α values for the Q_dl_ parameter differ significantly from unity. The constant phase element time constant is dispersed and Q_dl_ cannot be defined as capacitance. It is also worth noting that the highest α value is shown by the Q_dl_ parameter for the R2 sample, i.e., a sample containing a thick enough coating, thereby there is no need to take into account the use of the restricted linear diffusion element Ϻ.

**Figure 9:**
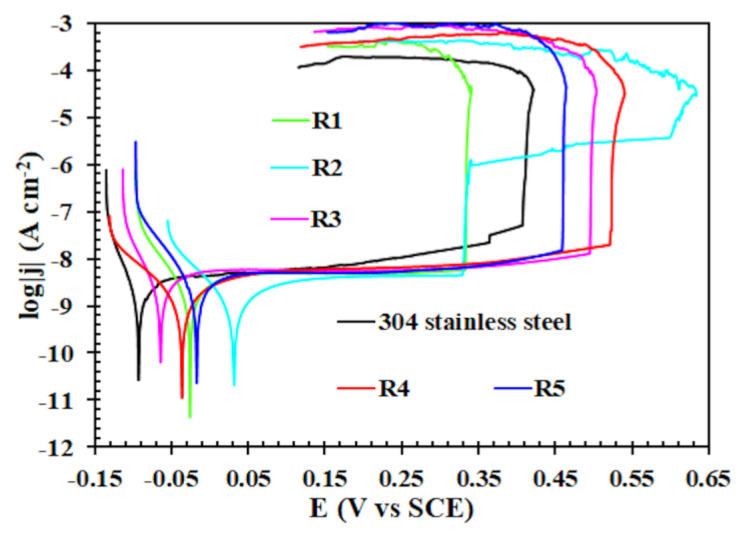
Potentiodynamic polarization curves of bare 304 stainless steel and samples R1–5.

Figure 9 shows the results of the potentiodynamic polarization (PP) tests, while Table 5 collects the values of corrosion potentials (E_corr_), corrosion current densities (j_corr_), slopes coefficients of cathodic (–β_c_) polarization curves and pitting potentials (E_pitt_) derived from these tests. The obtained results partly confirm the results of EIS measurements, i.e., sample R2 shows the highest value of E_corr_. Moreover, all samples whose surfaces were modified with siloxane coatings are characterized by higher E_corr_ values, i.e., they are more noble when compared to the unmodified 304 stainless steel. However, the samples with deposited siloxane coatings indicate higher values of j_corr_. As mentioned above, porous siloxane coatings are able to absorb the electrolyte solution, which implies the formation of conductive pathways inside these coatings. Therefore, most likely, the values of the corrosion current density are high. However, the values of individual samples are so small and similar to each other that it is difficult to find any reasonable relationship between the sol-gel solution preparation method and the j_corr_ of a given sample. The only regularity that appears is the fact that the sample on which the thickest coating has been deposited shows the lowest j_corr_. In the other samples, the presence of large, poorly soluble colloidal particles that make coatings thinner should be taken into account. Therefore, corrosion current density values become higher. This is due to the higher ratio of the absorbed amount of electrolyte to the thickness of the coating and also due to the presence/formation of corrosion products layer under the coatings. 

As mentioned earlier, the phase angle and impedance modulus values in the high-frequency range depend on the thickness and uniformity of the coating. This regularity can also be referred to the slope of the cathodic polarization curves. Taking into account that these coatings are porous, there is mass transport (electrolyte solution and oxygen) through their structure [45]. Therefore, it is concluded that these coatings do not completely insulate the steel surface. In addition, the tested systems are not under activation, but diffusive control. This applies to both polarization curves. The cathodic curve illustrates the oxygen reduction reaction. The slope of this curve indicates the extent of oxygen transport inhibition by a particular layer. Therefore, the slope of the cathodic polarization curve is an indicator of the thickness and uniformity of the coating. The highest slope values are characteristic for the R2 and R4 samples, i.e., the thickest and one of the thinnest (AFM) coatings, respectively. It follows that the thickness and uniformity of the coating have the same effect on the slope of the cathodic polarization curve. On this basis, it can be concluded that the coating on sample R4 is extremely uniform, which is confirmed by the results of EIS and potentiodynamic polarization tests. Unmodified steel indicates the lowest value of the curve slope. This is in line with expectations and is a reflection of the presence of a passive oxide film that is much thinner than coatings containing RPTMS.

Nevertheless, the greatest advantage of the used protective coatings is the inhibition of oxidation processes on the surface of 304 stainless steel. The phenomena of electrochemical corrosion of steel as well as metal elements include conjugated oxidation and reduction reactions (open circuit conditions). Both processes proceed on the surface of the corroding material and both of them affect the rate of the whole phenomenon. While the rate of electrochemical corrosion expressed by the value of corrosion current density for both modified and unmodified steel samples does not differ radically, when the oxidation reaction is inhibited, significant differences are noticed. Namely, for the samples modified with siloxane coatings the slope coefficients of the anodic (β_a_) polarization curves tend to infinity (β_a_→∞), while the slope coefficient β_a_ of bare steel is equal to ~1 V. Therefore, it is not necessary to include these values in Table 5 [52]. It is clear that the anodic process and therefore anodic polarization curves are under diffusive control. This is the diffusion of mass, but in this case, it concerns steel components (Fe, Cr, Ni) in the form of ions and hydroxides, which are weakly bonded to the steel surface [45,53]. Summarizing, this demonstrates the inhibition of the dissolution reaction of 304 stainless steel surface modified with siloxane coatings. This is also confirmed by the pitting potential values (E_pitt_). In the case of 304 stainless steel, E_pitt_ is equal to approximately 0.4 V vs SCE, which is in agreement with our previous studies [44]. Sample R1 is the only sample, which is characterized by a lower E_pitt_ value in comparison to unmodified steel. This is the sample that contains the thinnest coating. The highest E_pitt_ was obtained for sample R2, which is modified by the thickest and probably relatively uniform coating. This is indicated by all physicochemical, surface morphology and electrochemical studies, including potentiodynamic polarization. While the thickness of this coating is not disputed, one can argue about its uniformity. It is worth noting that the increase in current density already occurs at a potential of ~0.35 V vs SCE. However, a complete, uncontrolled increase in current density, corresponding to the pitting potential, occurs at a potential of 0.6 V, which is the highest value among all tested samples. The second highest value of E_pitt_ is indicated by sample R4, which contains one of the thinnest coatings. However, it can be assumed that it is a fairly uniform coating, which in addition to the high E_pitt_ value and EIS test results, also indicates a high |–β_c_| slope coefficient. It should also be mentioned that polarization tests, which aimed to determine the values of pitting potentials, are an excellent indicator of coating durability, i.e., they simulate an accelerated long-term test of coating durability in an aggressive, aqueous environment.

## 4. Conclusions

The organosilicon derivatives of rapeseed oil have been used to produce coatings protecting against steel corrosion. Due to the presence of alkoxysilyl groups and the use of sol-gel process, the obtained coatings are firmly bonded to the steel surface. All samples whose surfaces were modified with siloxane coatings were characterized by higher E_corr_ values, i.e., they are more noble when compared to the unmodified 304 stainless steel. The application of the silane solution of higher RPTMS concentration (containing TEOS) and shorter stirring time utilization permits the formation of thicker and more uniform coatings. The sample coated with the use of the solution containing higher RPTMS concentration and stirred for 3 h was characterized by the highest values of E_corr_ and E_pitt_. The greatest advantage of the protective coatings used was the inhibition of 304 stainless steel surface oxidation reactions. RPTMS based coatings demonstrate the capability of inhibition of the dissolution processes of 304 stainless steel surface.

## Figures and Tables

**Table 1:** Amounts of reagents used for the preparation of silane solutions.

Sample	Reagents [g]				
	RPTMS	Ethanol	Acetic acid	Water	TEOS *
R1	5	87	6	2	-
R2, R3 (72 h)	20	42	6	12	20
R4, R5 (72 h)	5	59	6	8	20

* TEOS: tetraethoxysilane.

**Table 2:** Thickness of the RPTMS layers.

Sample	Layer Thickness [nm]
R1	486 ± 31
R2	2030 ± 70
R3	920 ± 60
R4	613 ± 45
R5	800 ± 100

**Table 3:** Water contact angle (WCA) values of bare 304 stainless steel and samples R1–5.

Sample	WCA/Degree
304 stainless steel	70
R1	96
R2	96
R3	88
R4	94
R5	82

**Table 4:** Resistor (R), constant phase element parameter (Q) and restricted linear diffusion (R_d_, τ_d_) components values of different electric equivalent circuits (EEC) matched to EIS results of bare 304 stainless steel and samples R1–5.

Sample	Q_c_·10^−6^ (F s^(α−1)^)	α	R_c_ (kΩ)	R_d_ (Ω)	τ_d_ (s)	Q_dl_·10^−6^ (F s^(α−1)^)	α	R_ct_ (kΩ)
304 stainless steel	-	-	-	-	-	43.31	0.92	10,390
R1	0.84	0.83	43.40	15,193	0.94	11.18	0.54	-
R2	0.02	0.96	1,253	-	-	5.88	0.66	-
R3	0.58	0.84	13.58	5205	0.40	8.66	0.56	-
R4	0.39	0.81	17.20	70,124	6.54	24.53	0.56	-
R5	4.05	0.74	1.52	6.92	0.001	130.40	0.58	-

**Table 5:** Corrosion potentials (E_corr_), corrosion current densities (j_corr_), slopes of cathodic (β_c_) polarization curves and pitting potentials (E_pitt_) values for bare 304 stainless steel and samples R1–5.

Sample	E_corr_ (mV vs. SCE)	j_corr_ (nA cm^−2^)	−β_c_ (mV)	E_pitt_ (mV vs. SCE)
304 stainless steel	−93	3.80	40	410
R1	−26	5.00	74	335
R2	32	4.30	125	600
R3	−65	6.00	59	495
R4	−36	6.10	165	520
R5	−17	4.90	60	460

## Supplementary Materials

The following are available online at https://www.mdpi.com/1996-1944/13/10/2212/s1, Figure S1: EDS mapping for all coating.
