# Supplementary material for: The Rapeseed Oil Based Organofunctional Silane for Stainless Steel Protective Coatings
Source: Materials (Basel). 2020 May 12;13(10):2212. doi: 10.3390/ma13102212 (PMC7287667; doi:10.3390/ma13102212)
Supplement: Supplementary file 1 [file materials-13-02212-s001.pdf]

Supporting Information

The rapeseed oil based organofunctional silane for stainless steel protective coatings

Karol Szubert, Jarosław Wojciechowski, Łukasz Majchrzycki, Wojciech Jurczak, Grzegorz Lota and Hieronim Maciejewski

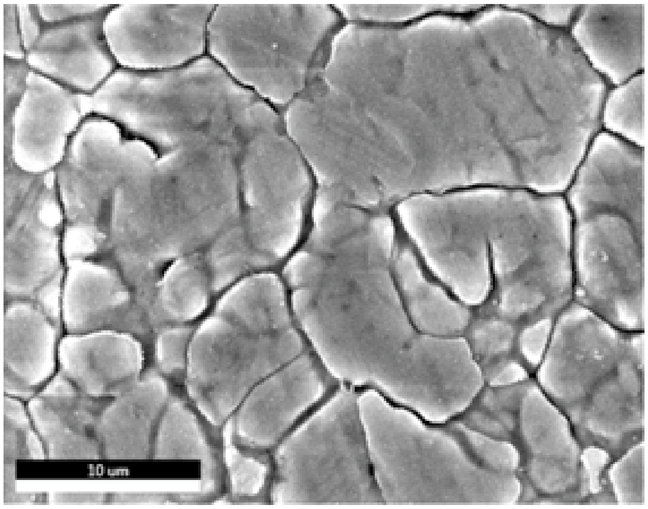

Back-scattered electron image of 304 stainless steel after acetone and hot 10% KOH solution treatment.

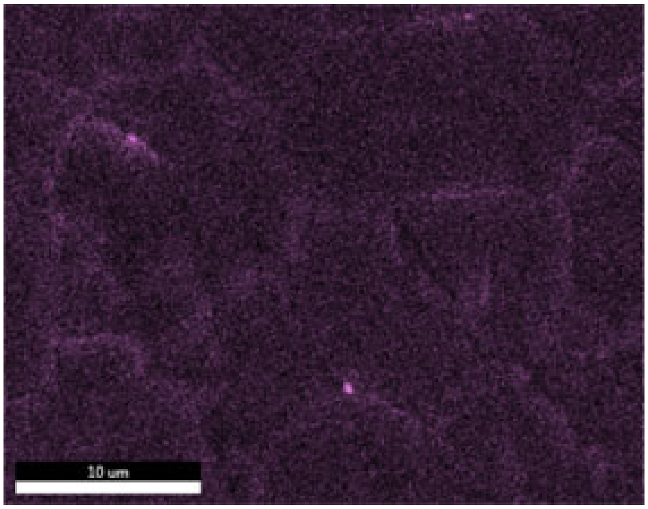

Element map of Si in 304 stainless steel after acetone and hot 10% KOH solution treatment.

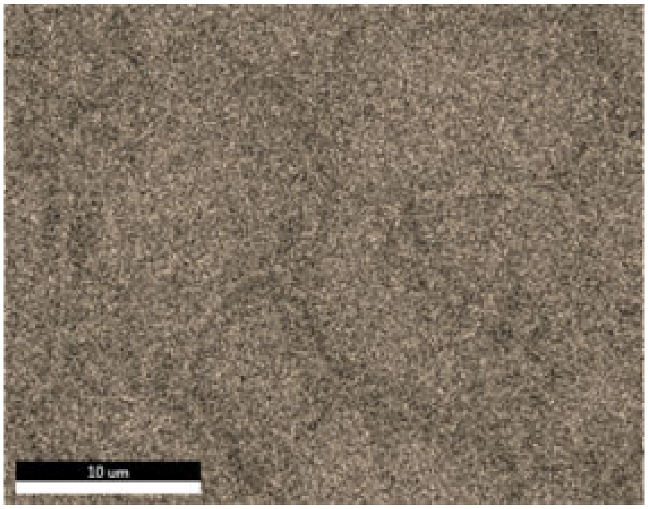

Element map of Cr in 304 stainless steel after acetone and hot 10% KOH solution treatment.

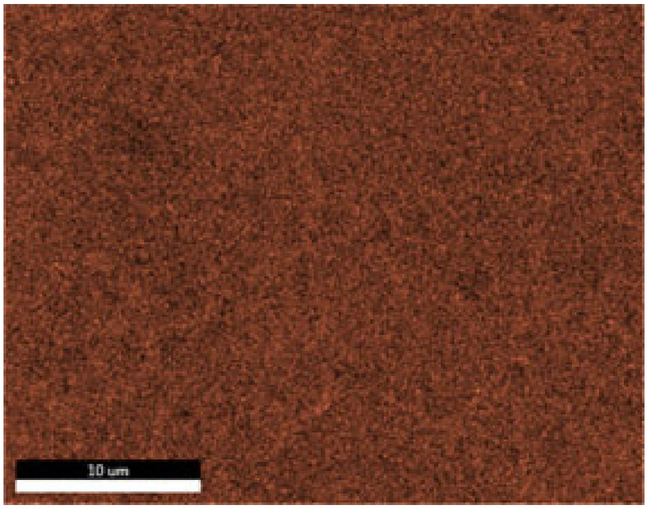

Element map of Fe in 304 stainless steel after acetone and hot 10% KOH solution treatment.

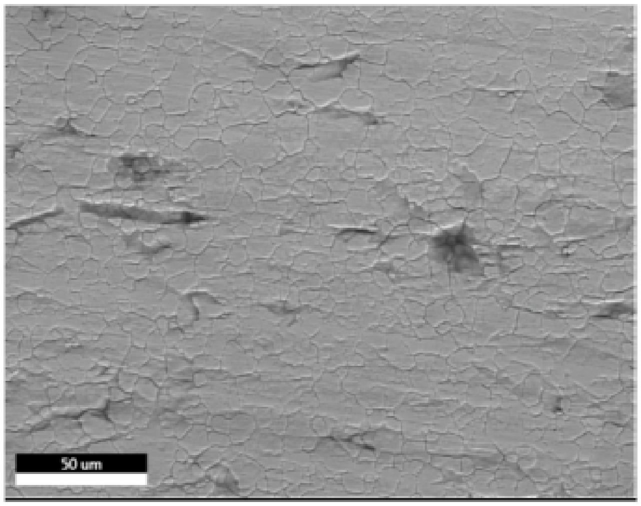

Back-scattered electron image of RPTMS-coated samples **R1.**

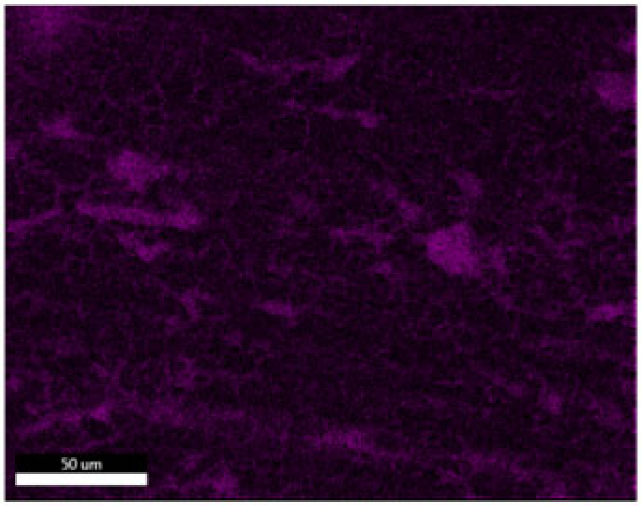

Element map of C in RPTMS-coated samples **R1.**

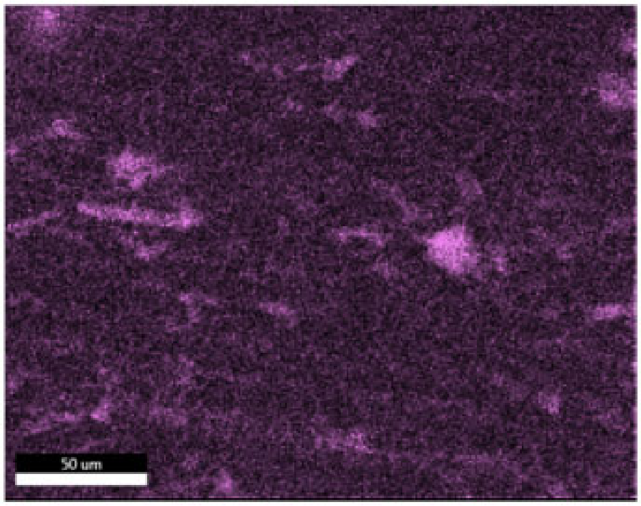

Element map of Si in RPTMS-coated samples **R1.**

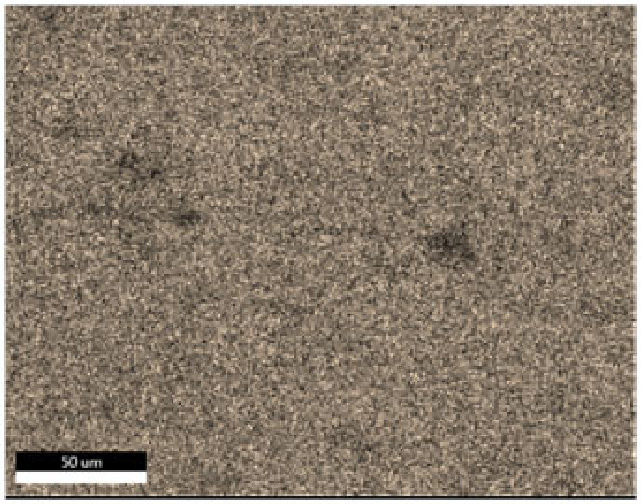

Element map of Cr in RPTMS-coated samples **R1.**

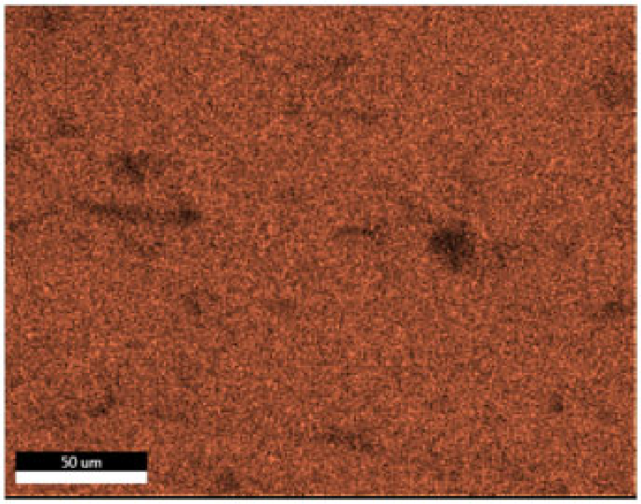

Element map of Fe in RPTMS-coated samples **R1.**

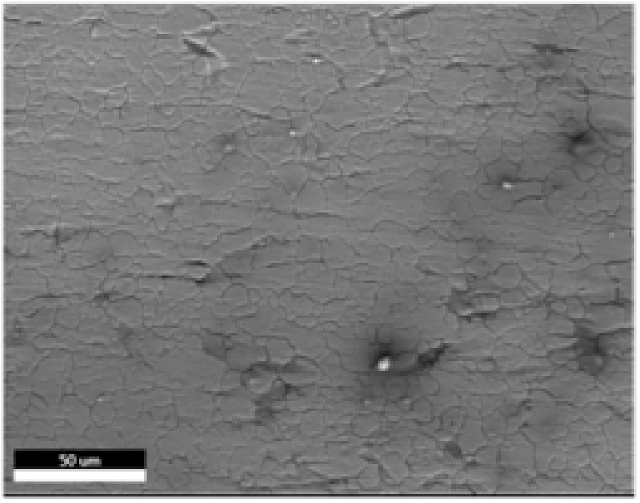

Back-scattered electron image of RPTMS-coated samples **R2.**

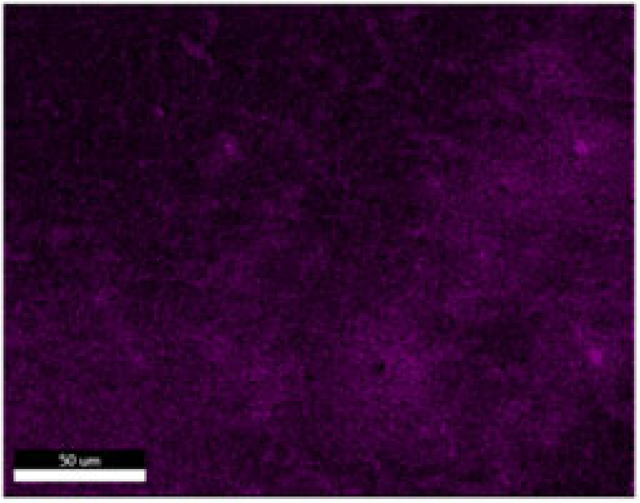

Element map of C in RPTMS-coated samples **R2.**

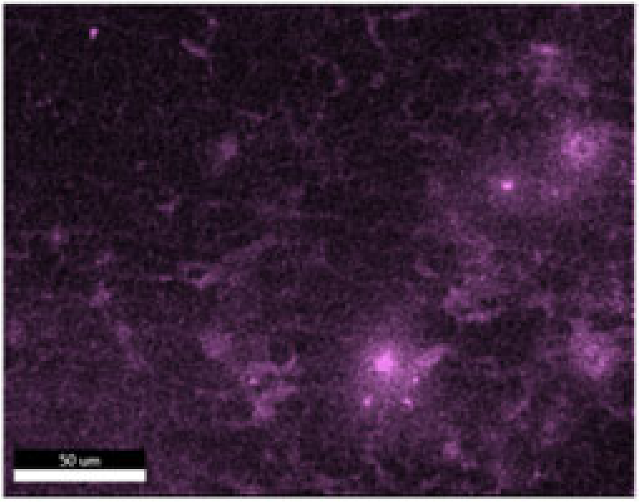

Element map of Si in RPTMS-coated samples **R2.**

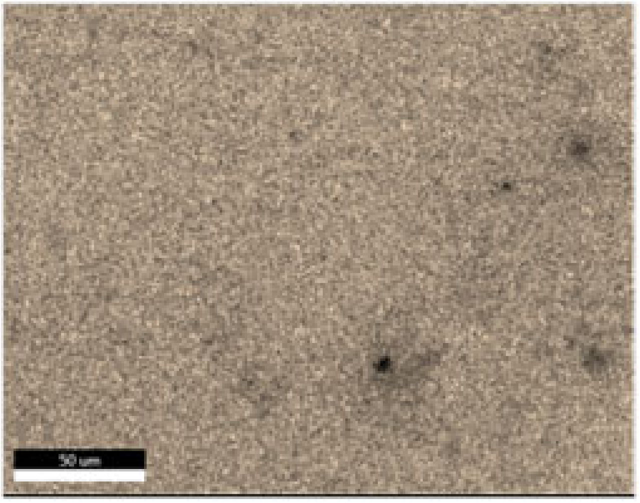

Element map of Cr in RPTMS-coated samples **R2.**

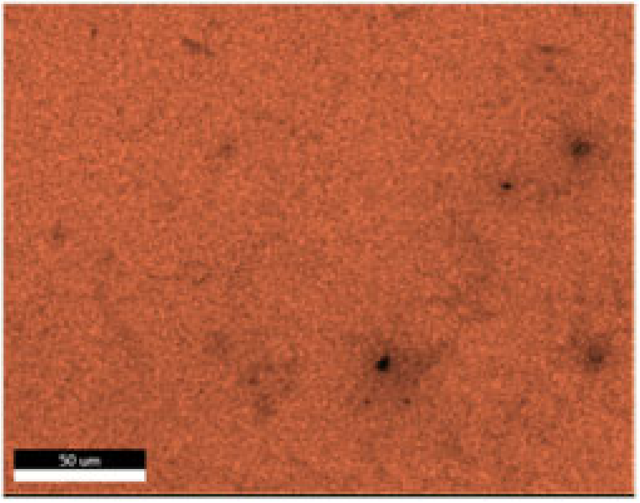

Element map of Fe in RPTMS-coated samples **R2.**

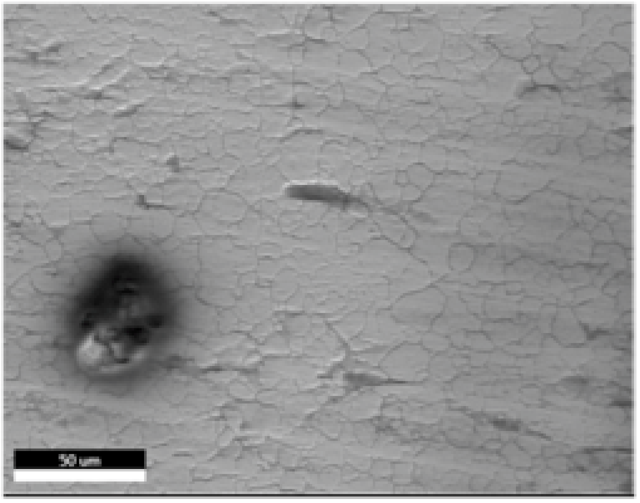

Back-scattered electron image of RPTMS-coated samples **R3.**

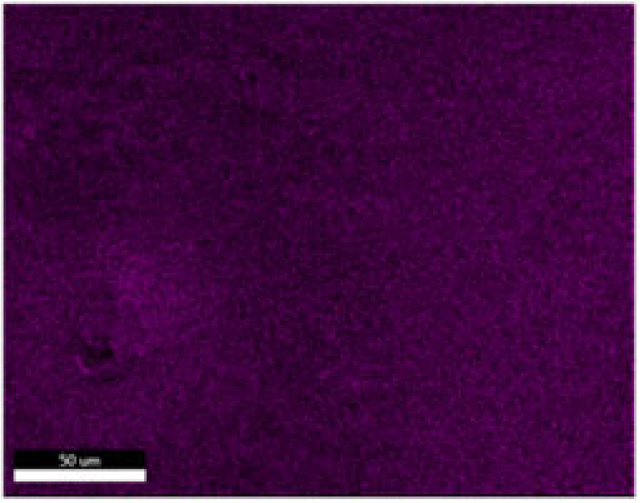

Element map of C in RPTMS-coated samples **R3.**

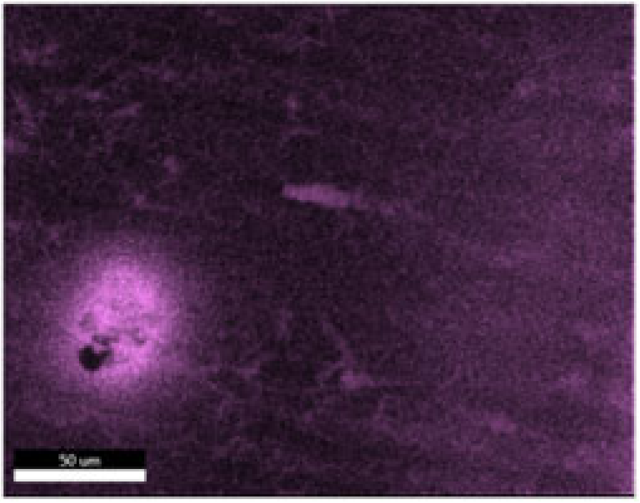

Element map of Si in RPTMS-coated samples **R3.**

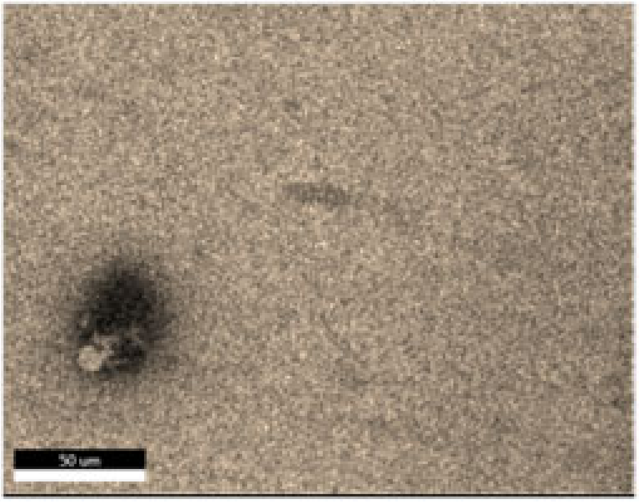

Element map of Cr in RPTMS-coated samples **R3.**

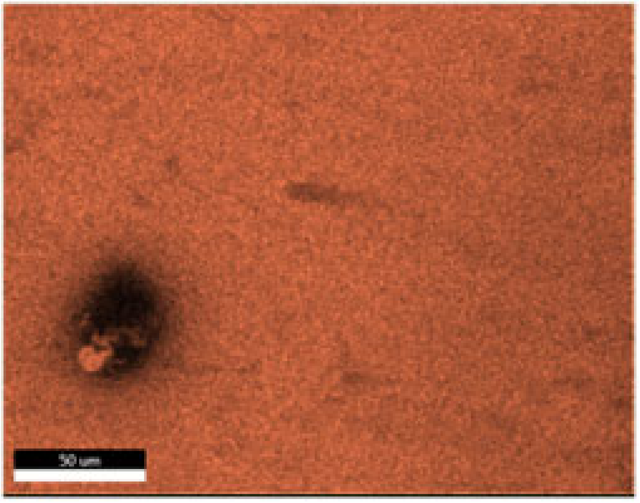

Element map of Fe in RPTMS-coated samples **R3.**

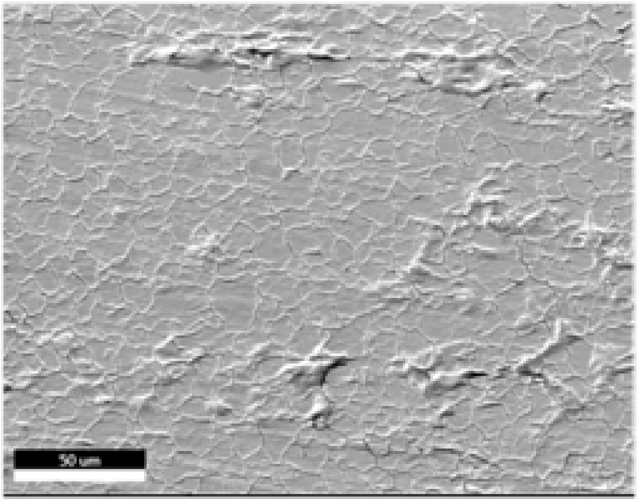

Back-scattered electron image of RPTMS-coated samples **R4.**

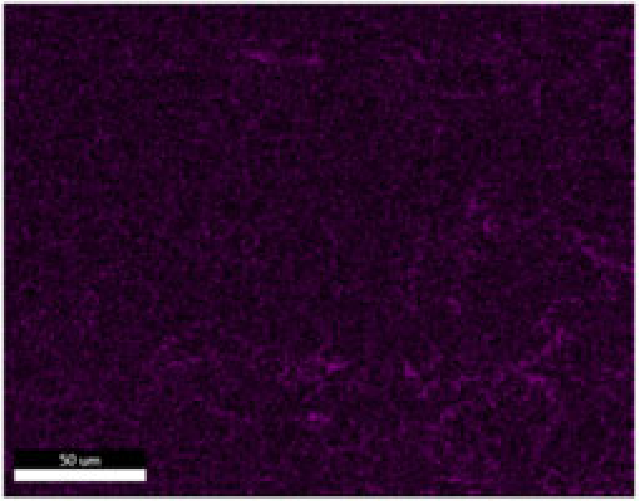

Element map of C in RPTMS-coated samples **R4.**

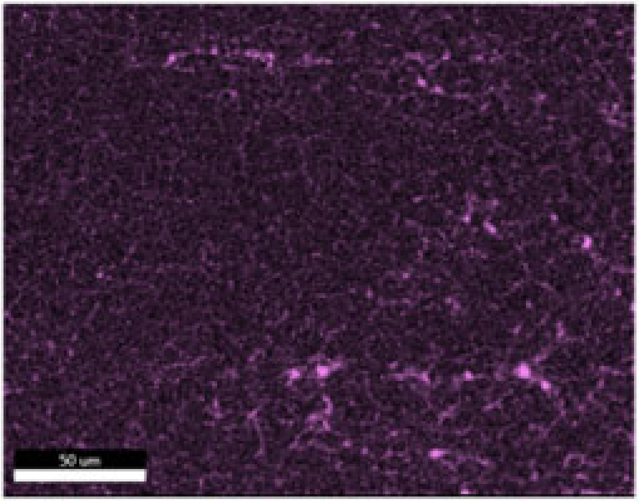

Element map of Si in RPTMS-coated samples **R4.**

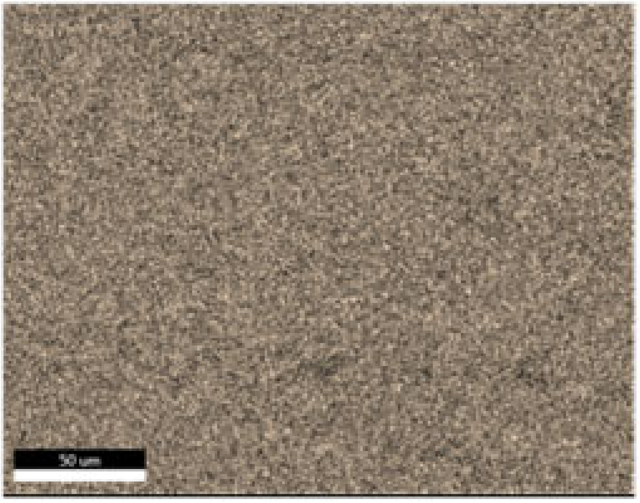

Element map of Cr in RPTMS-coated samples **R4.**

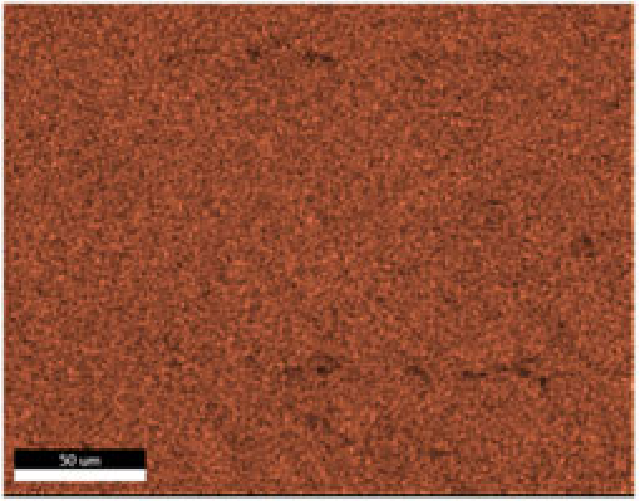

Element map of Fe in RPTMS-coated samples **R4.**

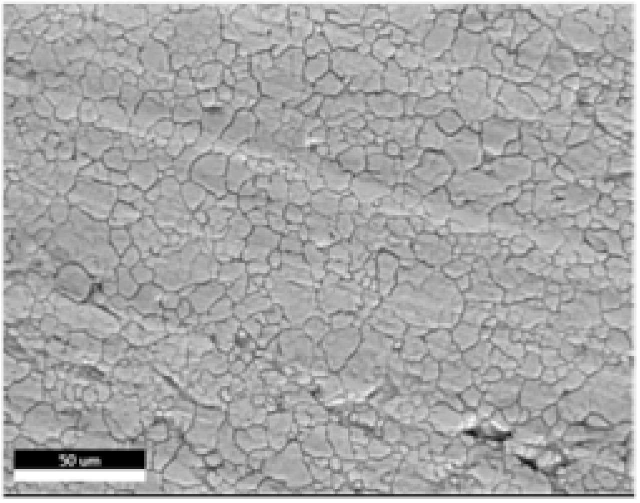

Back-scattered electron image of RPTMS-coated samples **R5.**

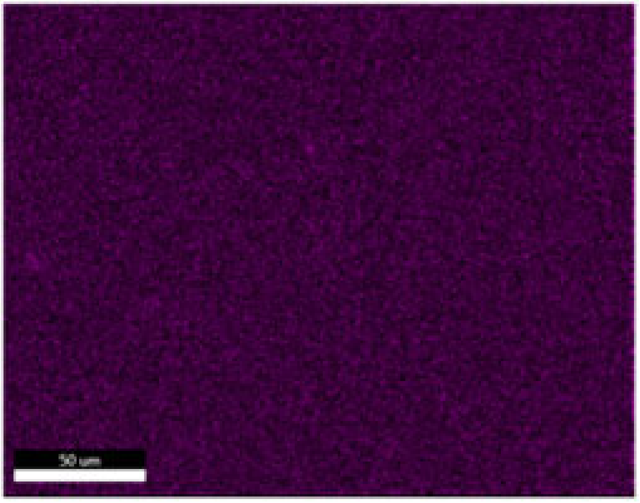

Element map of C in RPTMS-coated samples **R5.**

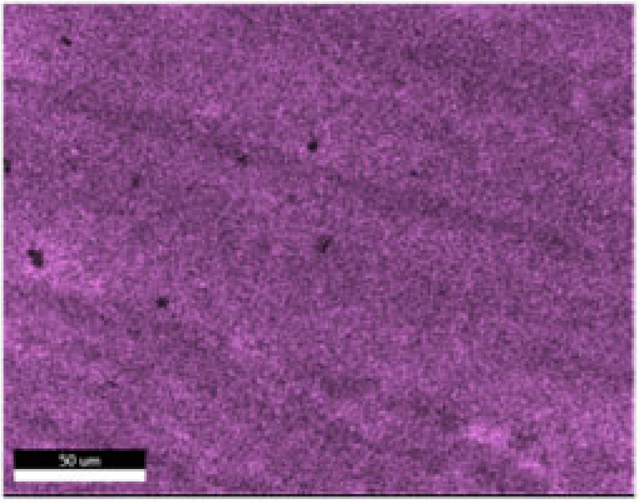

Element map of Si in RPTMS-coated samples **R5.**

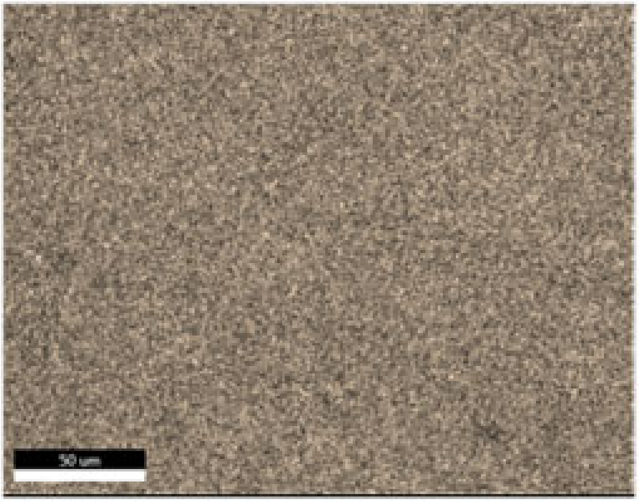

Element map of Cr in RPTMS-coated samples **R5.**

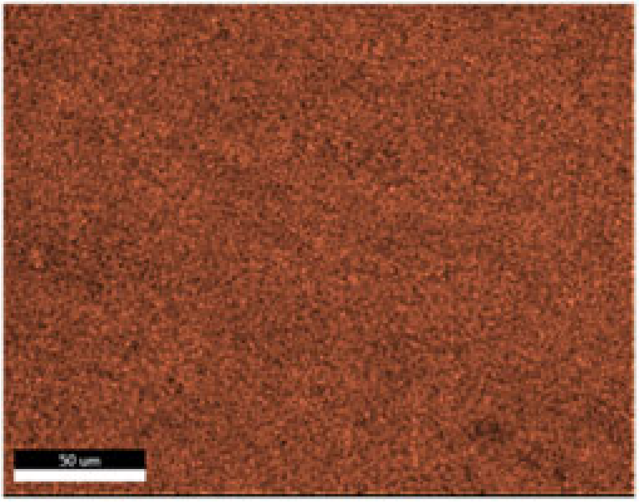

Element map of Fe in RPTMS-coated samples **R5.**

Figure S1. EDS mapping for all coating.
